# A network analysis of housing quality indicators and depression in women

**DOI:** 10.1038/s41598-025-22353-z

**Published:** 2025-11-05

**Authors:** Faye Sanders, Lucy H. Waldren, Vilte Baltramonaityte, Alexandre A. Lussier, Esther Walton

**Affiliations:** 1 Department of Psychology, University of Bath, Bath, BA2 7AY UK; 2 Psychiatric and Neurodevelopmental Genetics Unit, Center for Genomic Medicine, Massachusetts General Hospital, Boston, MA United States; 3 Department of Psychiatry, Harvard Medical School, Boston, MA United States; 4 Stanley Center for Psychiatric Research, The Broad Institute of Harvard and MIT, Cambridge, MA England

**Keywords:** Public health, Environmental social sciences

## Abstract

Numerous studies have detected associations between poor housing quality and increased risk for mental illness. However, it currently remains unclear in associations between poor housing quality and increased risk for women’s mental illness which housing quality indicators drive this association and hence which specific indicators should be prioritised in housing quality assessments or improvements. In a sample of up to 9,669 pregnant women, we used a network analysis to investigate cross-sectional associations between poor housing quality indicators (e.g., house size, facilities, leaks or condensation/mould, decorations, and feelings towards the home) and depressive symptoms (assessed at age 28). All 36 edges showed non-zero associations, whereby when considering all poor housing quality indicators ‘feelings-towards-the-home’ had the strongest association with depressive symptoms, and ‘feelings-towards-the-home’, in turn, was most strongly associated with house problems, size, and facilities. Our findings highlight the importance of using multiple (or composite) person-centred measures of housing quality in the context of maternal mental health.

**Supplementary Information:**

The online version contains supplementary material available at 10.1038/s41598-025-22353-z.

## Introduction

Individuals living in poor housing conditions can be up to eight times more likely to experience depressive symptoms or anxiety, and this risk has been shown to persist over a follow-up period of up to 32 years^1–4^. Strong evidence also suggests these associations are not limited to contexts of lower socioeconomic status and are not solely driven by pre-existing mental health^3^. The importance of housing quality for maternal mental health, including depression and anxiety, is well supported^5,6^. For example, mothers who lived in dark, crowded or noisy homes were almost 1.5 times more likely to screen positive for depression even after adjusting for other social stressors and financial hardship^5^. In addition, mothers who experience a housing crisis, including eviction or homelessness, are at significantly more risk of experiencing a depressive episode within 12 months of the crisis^6^. Research also indicates both women and older adults are at greater risk, than men and younger counterparts, of experiencing poor mental health associated with poor housing quality^7^. Hence, with this growing recognition of poor housing quality as a risk factor for maternal mental health^3,4,7^, it becomes increasingly important to translate housing research into actionable outcomes for population health.

However, measurements of poor housing quality often vary across the literature. For example, house size, instability, and cleanliness are all examples of housing quality measurements used in associations between housing and mental health^2,8–10^. Further, some studies have used house-centered measures of housing quality, such as the number of rooms or temperature in a home^2,8^. By contrast, others have focussed on person-centered measures of housing quality, such as feelings of safety or reported problems in the home^11,12^. Whilst this body of literature demonstrates the complexity of housing quality and its association to mental health, the lack of standardisation makes it challenging to compare results and identify which features of housing quality are most important for mental health.

More recently, studies have used composite scores of multiple poor housing quality indicators^2,3,7^, as these scores might better capture the complexity of housing quality and its relationship with health. However, composite scores can hide the individual contributions of housing indicators to associations between housing and health. It hence remains poorly understood which specific housing quality indicators primarily drive associations with mental health. This limitation becomes particularly important when considering the potential applications of this research towards housing policies, where prioritisation of certain housing quality indicators may be needed due to cost constraints.

Most studies have used regression models with a single predictor to assess associations between poor housing quality and mental health^3,4,7^. Although such methods can assess the relationship between poor housing quality and mental health, they cannot investigate the complexity of poor housing quality itself, including how specific poor housing quality indicators relate to each other (and to mental health). More novel methods, such as network analyses^13^, can investigate relationships between variables and the individual contributions of each variable to a network. By taking a step back from a classic single-predictor regression approach, network analysis can ascertain which variables have the largest influence in the network as a whole.

This study set out to address the above challenges, including uncertainties surrounding person- versus house-centered housing measures as risk factors for depression and how housing quality indicators relate to each other in women. We investigated the network of individual housing quality indicators, which we previously used within a composite housing quality score^3^, and their relative contributions in the association between poor housing quality and depressive symptoms.

## Results

### Descriptive statistics

All participants were female and aged between 15 and 44 years at enrolment (Table 1). 22% had a university degree and an additional 30% completed their A-levels. The average number of times moved in the last 5 years at the beginning of the study was 1.65 (*SD* = 1.75).

**Table 1:** Study descriptives for 9,669 women who had completed questionnaire items for their depressive symptoms at enrolment (imputed data).

**Poor** **Housing Quality**	
Mean (SD)	3.44 (0.34)
Min, Max	1.21, 4.81
Depressive Symptoms ^1^	
Mean (SD)	4.30 (3.02)
Min, Max	0.00, 16.00
**Age (years) **	
Mean (SD)	28.36 (4.67)
Min, Max	15.00, 44.00
Ethnicity ^2^	
White	98.12%
Non-White	1.88%

^1^ Depressive symptoms used the Crown Crisp Experiential Index.

^2^ Ethnicity (e.g., black/Caribbean, black/African, black/other, Indian, Pakistani, Bangladeshi, Chinese, any other ethnic group) was self-reported at study enrolment.

SD = standard deviation.

**Fig. 1:**
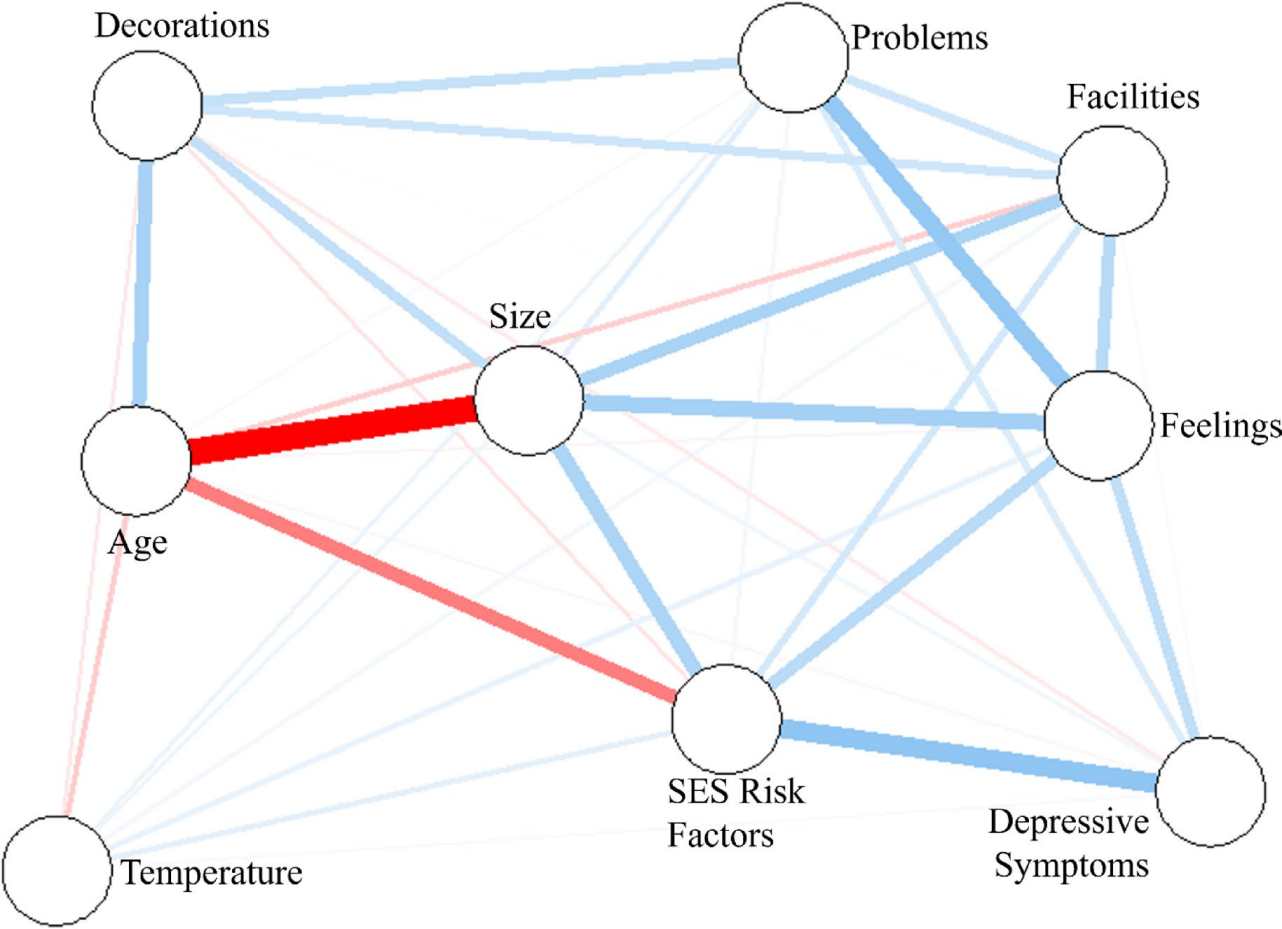
Network analysis of poor housing quality indicators with depressive symptoms in women, including socioeconomic status (SES) risk factors and age (at depressive symptoms). Circles (nodes) represent variables and lines (edges) represent the relationships between variables. Line thickness represents the strength of edges, and line colour (blue = positive, red = negative) represent the directionality of the edge.

All 36 possible edges showed nonzero associations with a mean edge weight of 0.04 (SD = 0.10; range=−0.30 to 0.21, within a possible range of −1.0 to 1.0; Fig. 1; Table S2), indicating a complex network between poor housing quality indicators and depressive symptoms.

**Fig. 2:**
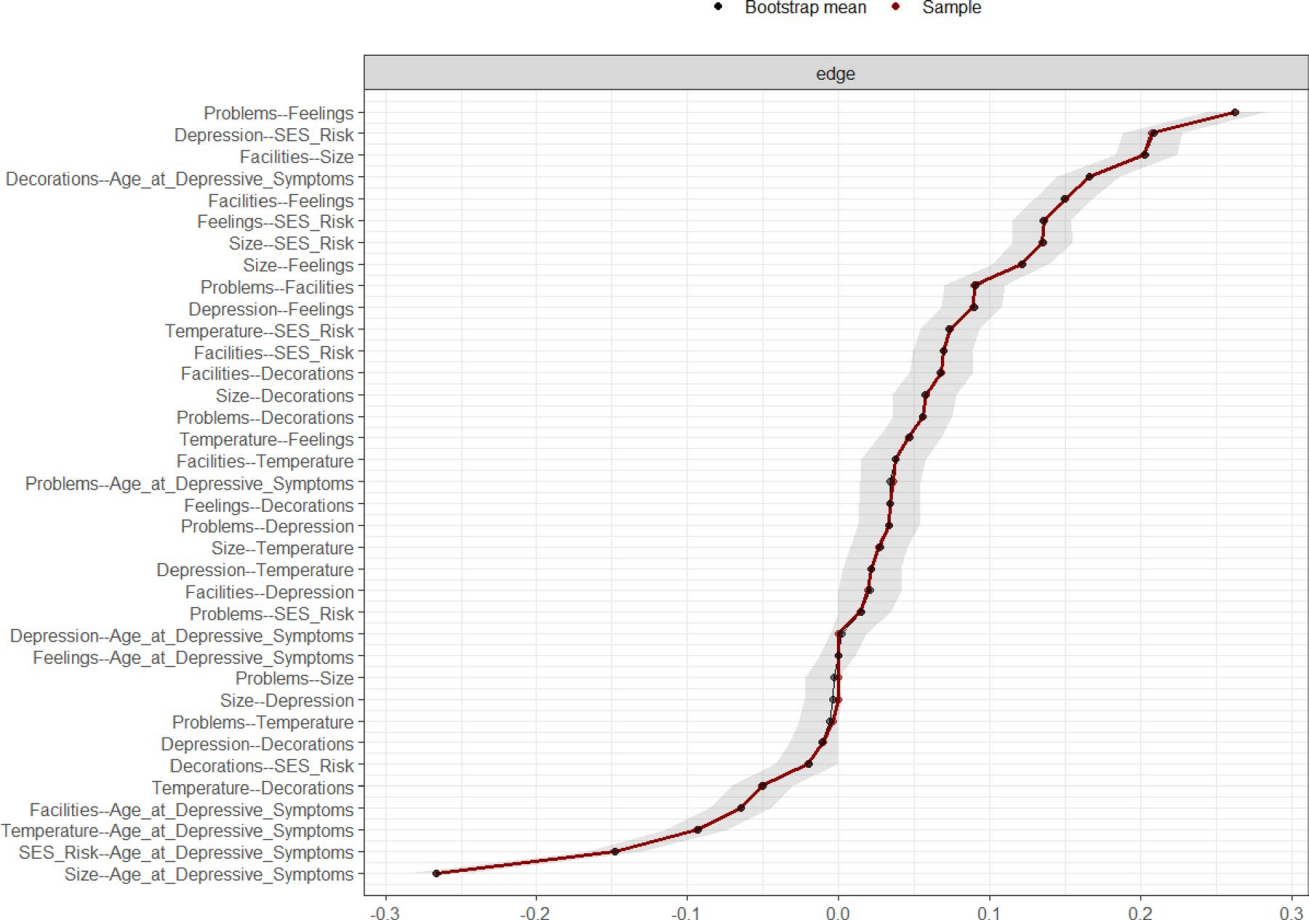
Estimated edge weight accuracy assessed through non-parametric bootstrapping (1,000 resamples). The sample edge weights are shown in red and the bootstrapped edge weights are shown in black. Shaded areas represent 95% bootstrapped confidence intervals.

**Fig. 3:**
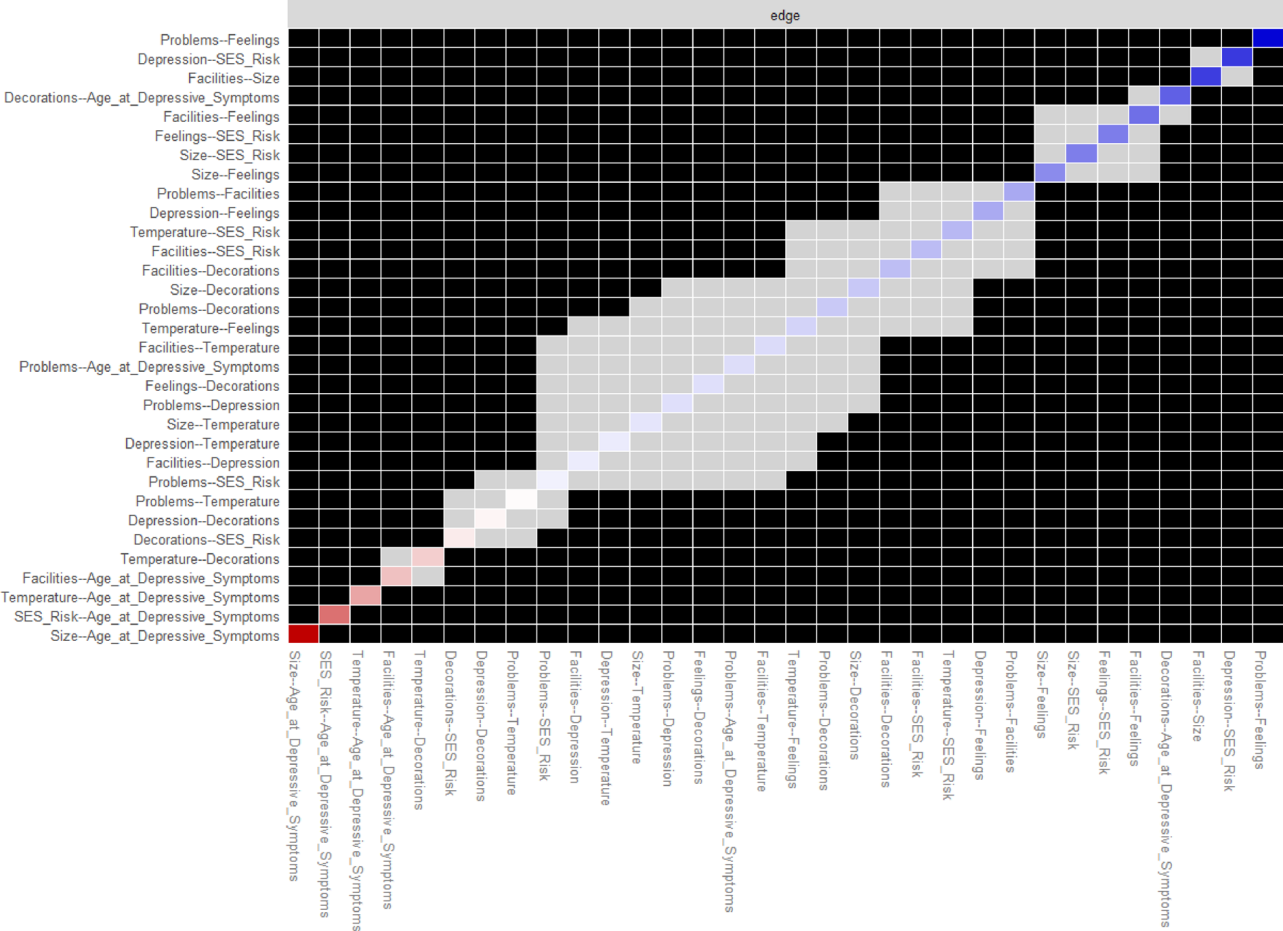
Plot of edge weight difference test using 95% confidence interval bootstrapping with 1,000 resamples. Black squares represent significant differences between the edge weights, and grey squares represent no significant differences. Significant differences are tested by comparing the observed edge weights to the distribution of bootstrapped values. In other words, black squares represent the most robust edges in the network. The edges with the strongest associations are blue, with weakest associations in pink.

Nonparametric bootstrapping showed that edge weights were determined with acceptable accuracy, evidenced by the small variation in edge weight estimates across 1,000 bootstrap samples, with 95% confidence intervals (Fig. 2). We identified the two strongest overall edges (associations) between (smaller) house size and age (−0.301) and depressive symptoms and SES risk (0.205). When examining edges among non-covariate nodes, we identified the strongest edges between feelings towards the home and house problems (0.197), house size and feelings (0.166), and house size and facilities (0.157). Of all six housing quality measures, depressive symptoms was most strongly linked to feelings (0.116) (Fig. 2; Table S2). These edges were significantly greater in strength (*p* < .05) than other edges in the network, as identified in the edge weight difference test (Fig. 3).

Case-drop bootstrapping suggested measures of closeness (Coefficient of Stability, CS = 0.89), expected influence (CS = 0.89, and betweenness (CS = 0.89) far exceeded the minimal levels of acceptance for network models (CS > 0.25) (Figure S1). SES Risk (0.61), depressive symptoms (0.36) and feelings towards the home (0.26) had the greatest expected influence, and house decorations (0.11) had the least expected influence across the network (Figure S2; Table S3). House decorations had the greatest betweenness score (6.0; Table S3), meaning it was a node that often laid along the shortest path of other nodes.

## Discussion

We investigated associations between six poor housing quality indicators and depressive symptoms using a network analysis. We identified feelings towards the home as a key variable in the network, which of all poor housing quality indicators, showed the strongest associations with house problems, followed by house size and depressive symptoms. We highlight and discuss three main findings.

First we found feelings towards the home, a person-centered measure of housing quality, had the greatest association with depressive symptoms in women than any other housing quality indicator. Whilst previous studies have evidenced the importance of housing for women’s mental health in particular^5–7^, our findings extend this work by providing more fine-grained evidence on specific features of housing important for maternal mental health. For example, our findings show subjective feelings towards the home may be especially central to maternal mental health, above and beyond previous physical qualities associated with maternal mental health, such as darkness and noise^5^. This finding is particularly important, as currently most studies on housing and health typically rely on house-centered measures, such as house size or dampness and mould^2,4^. Similarly, the UK Decent Homes Standard – a government scheme outlining the minimum levels of quality that social housing must meet – does not cover any person-centered measures of housing quality^14^. Whilst depressive symptoms likely have reverse-causal relationships with feelings towards the home, we previously determined that controlling for baseline depressive symptoms does not diminish associations in this study population^3^. Hence, our findings suggest that including person-centered measures focussed on feelings towards the home could substantially improve current assessments of housing quality in the context of maternal mental health.

Second, feelings towards the home was most strongly associated with problems within the home, including damp, condensation, mould, and leaks. This finding emphasises how subjective attitudes towards the home may be most strongly determined by problems that can have severe health consequences, such as mould. Although targeting subjective attitudes towards the home may seem challenging, this study demonstrates how focussing on serious problems in homes could potentially approximate subjective measures. Feelings towards the home were also strongly associated with house size. This association is consistent with prior literature that identified associations between overcrowding and decreased well-being^15^.

Third, most housing quality indicators had positive associations with each other, suggesting features of housing quality co-occur. For example, there was a positive association between house size and facilities. This result suggests homes of greater sizes were more likely to contain more housing facilities, such as outside spaces. However, decorations of the home had an unexpected weak but negative association with housing temperature. This finding demonstrates how poor housing quality indicators do not always align with each other.

Our findings should be considered along with their limitations. Firstly, this network analysis was only conducted in adult women. Whilst we included SES risk factors and age in the network, we did not test whether network associations between different housing quality indicators and depressive symptoms vary by gender. However, focusing specifically on mothers also offers a unique strength by allowing for clearer understanding of the role of housing in maternal mental health. Secondly, our network analysis investigated cross-sectional associations between housing quality indicators and total depressive scores. As depression is a heterogenous condition with varying symptom profiles and trajectories^16^, its associations with housing quality indicators might vary by symptoms and across time. Thirdly, this network analysis is not able to provide insights into the directionality of associations detected. We previously showed that reverse-causation is unlikely; patterns of associations between *overall* poor housing quality and depressive symptoms remained largely consistent when controlling for baseline depressive symptoms in a longitudinal study in the same study population^3^. However, the directionality between *individual* housing quality indicators remains more unclear.

To conclude, we found feelings towards the home to be an important variable in the network and it was most strongly associated with house problems, size, and depressive symptoms. The complexity of the network evidences the importance of considering multiple indicators or composite scores as measures of housing quality, as they provide comprehensive insights into the role of housing quality in mental health. The variability in associations between specific housing quality indicators and depression specifically supports indicator-specific analyses to reveal associations initially hidden by composite scores. Our findings also highlight the importance of considering person-centered measures of housing quality when addressing poor housing quality as a potential policy target for improving population mental health.

## Methods

### Participants

Participants were mothers from the Avon Longitudinal Study of Parents and Children (ALSPAC) cohort^17–19^. Pregnant women resident in Avon, UK with expected dates of delivery 1 st April 1991 to 31 st December 1992 were invited to take part in the study and the initial number of pregnancies enrolled was 14,541. We analyzed the 9,669 mothers who had completed at least 50% of questionnaire items for their housing circumstances at enrolment. In that sample of 9,669 mothers, remaining missing data for poor housing quality and depressive symptoms were imputed using the MICE R package (^20^ see supplementary materials Sect. 1.1 for further information). We conducted a missing-at-random (MAR) analysis as reported in Table S1. All methods were performed in accordance with relevant guidelines and regulations.

## Measures

### Poor housing quality

**Fig. 4:**
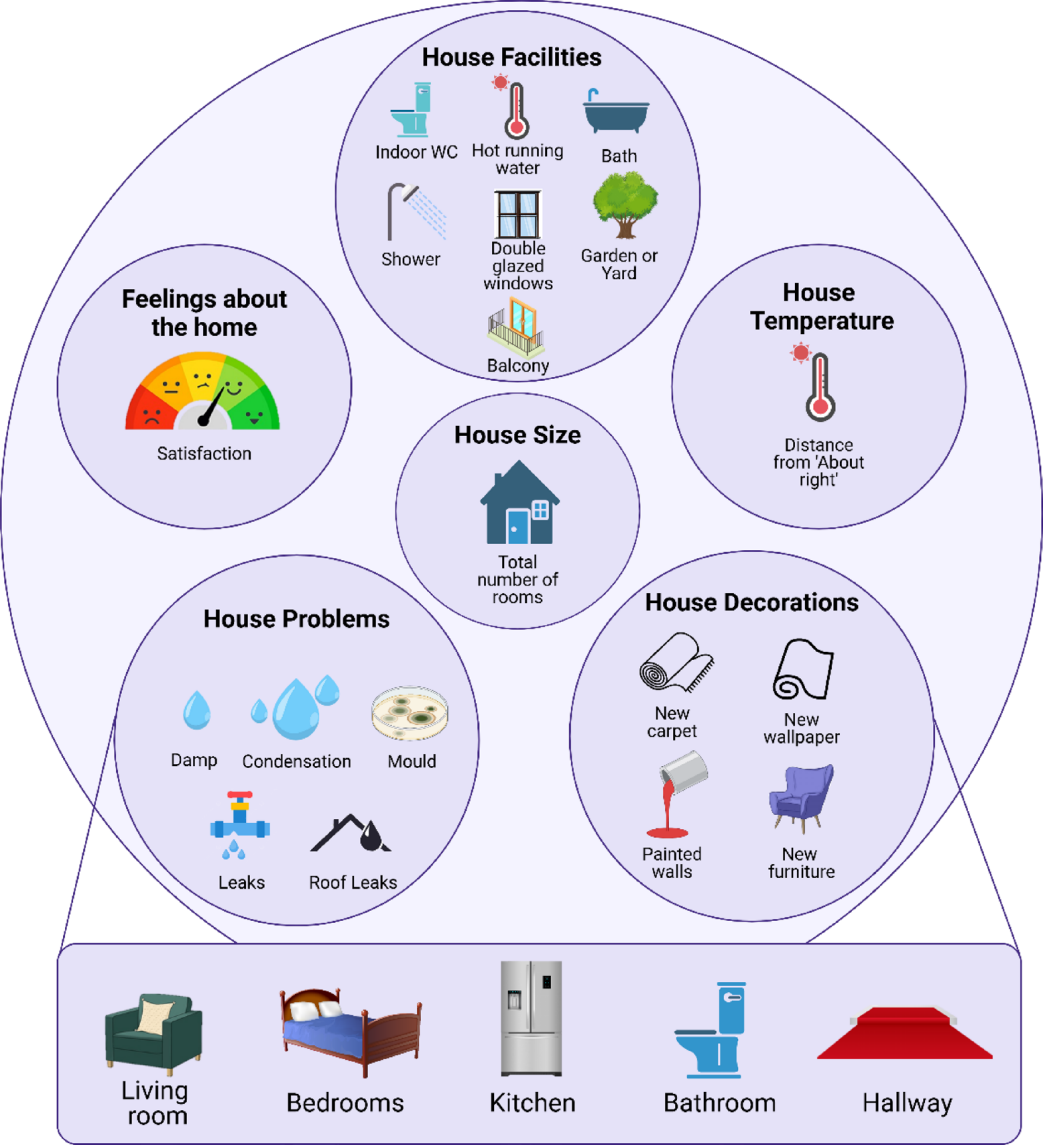
Six ‘Poor Housing Quality’ indicators used in the network analysis^3^.

As described in detail in Sanders et al.^3^ , general housing data of six key indicators (e.g., size, temperature, facilities, decorations, problems, and feelings towards the home; Fig. 4) were collected using self-report questionnaires completed by the mothers at study enrolment (mean age 28 years). These indicators include both person-centered and house-centered variables in housing. More information can be seen in supplementary materials Sect. 1.2.

### SES risk

SES risk factors scores – comprised of mother’s education, experience of reduced income, losing a job, becoming homeless, and financial difficulties – were measured at study enrolment, 1- and 2-year follow-ups. Education was coded, in order of highest to lowest SES risk factors, as ‘CSE’, ‘Vocational’, ‘O level’, ‘A level’, and ‘Degree’. Experiences of reduced income, becoming homeless and financial difficulties, in order of highest to lowest SES risk factors, were coded as ‘Affected a lot’, ‘Moderately affected’, ‘Mildly affected’, ‘No effect at all’, and ‘Did not happen’.

### Depressive symptoms

Depressive symptoms were measured at study enrolment using the Crown Crisp Experiential Index (CCEI)^21^. The CCEI is a commonly-implemented measure of depression that comprehensively assesses features of depression including anhedonia and sadness. The CCEI consisted of 10 items such as, ‘I have felt sad or miserable’ and ‘I have been so unhappy that I have been crying’, with responses including ‘Yes, most of the time’, ‘Yes, quite often’, ‘Not very often/Only occasionally’ and ‘No, not at all/No, never’. Scores can range from 0 to 30.

### Statistical analyses

To investigate the contribution of individual poor housing quality indicators to depressive symptoms in women, a weighted, non-directional network was created. In network analysis, variables are represented by nodes (circles), and the relationships between variables are represented by edges (connecting lines). Because each edge accounts for all other edges in the network, this method can determine the strength of associations within the network and the importance of each node in the network. Here, each node represented one housing quality item (e.g., house facilities) or composite depressive symptoms, while each edge represented the unique conditional relationship between two housing items or one housing item and depression. The network was adjusted for age and SES risk. As a missing-at-random analysis indicated the presence of some bias (Table S1), we performed inverse probability weighting. A binary selection indicator (S) was coded as 1 if all pre-specified baseline housing variables were complete and 0 otherwise. After applying the inclusion criteria (< 50% missingness on housing measures) to the imputed dataset, we estimated the probability of a non-missing observation using logistic regression with baseline age (at depressive symptoms assessment) and SES risk factors as predictors. Stabilized weights were computed as the marginal probability of observation divided by each participant’s predicted probability, and these weights were incorporated into the weighted Spearman correlation matrices used for network estimation.

To perform our network analysis, we used the qgraph package^22^ in R Studio Statistical Software (v4.1.0; R Core Team, 2021), which uses a conservative EBICglasso and Gaussian Graphical Models using partial coefficients. A moderate EBIC tuning parameter of 0.5 was selected as the penalty to generate a network that excludes weak edges to reduce noise, whilst still retaining sufficient sensitivity to detect important edges^23^.

We performed nonparametric case-drop bootstrapping (1,000 resamples), with 95% confidence intervals (CIs), to assess the accuracy of the network’s edge weights. This analysis involves removing a subset of edges randomly from the network multiple times to create new “bootstrap” samples^22^. We then performed edge weight difference tests to identify the greatest edges in the network.

## Supplementary Information

Below is the link to the electronic supplementary material.

Supplementary Material 1

## Data Availability

Please note that the study website contains details of all the data that is available through a fully searchable data dictionary and variable search tool ([http://www.bristol.ac.uk/alspac/researchers/our-data/](http:/www.bristol.ac.uk/alspac/researchers/our-data)). Access to ALSPAC data is through a system of managed open access ([http://www.bristol.ac.uk/alspac/researchers/access/](http:/www.bristol.ac.uk/alspac/researchers/access)).
