# Supplementary material for: A network analysis of housing quality indicators and depression in women
Source: Sci Rep. 2025 Nov 5;15:38745. doi: 10.1038/s41598-025-22353-z (PMC12589598; doi:10.1038/s41598-025-22353-z)
Supplement: Supplementary file 1 — Supplementary Material 1 [file 41598_2025_22353_MOESM1_ESM.docx]

**Supplementary Materials**

A Network Analysis of Housing Quality Indicators and Depression in Women

**1 Methods**

- 1. **Missing Data Imputations**

As described in Sanders et al. ^1^, mothers who had completed at least 50% questionnaire items for their housing circumstances at study enrolment, leaving 9,669 mothers available for analysis. Missing data for the remaining poor housing quality, depressive symptoms, and a number of auxiliary variables were imputed. Imputation was conducted using the R MICE package ^2^. Poor housing quality and all auxiliary variables were used as predictors in a predictor matrix to impute missing data (in poor housing quality, all auxiliary variables and depressive symptoms. Auxiliary variables included SES risk factors, familial depressive history, poor neighbourhood quality, age (at depressive symptoms measurement), smoking, ethnicity, age at blood sample assessment, age at housing and housing instability. Age at blood sample assessment was initially included as an auxiliary variable due to its relevance in our previous study ^1^.

Imputation was performed using 60 iterations and 30 imputed datasets. The predictor matrix was specified using *quickpred*, with a minimum correlation set to 0.05, striking a balance between having sufficient variable breadth yet avoiding weak predictors. The predictor matrix was modified so depressive symptom scores would be imputed but not used as predictors.

We compared demographics between those who had >50% missing data for poor housing quality at study enrolment (n = 4702) and those who did not (i.e., analytic sample; N = 9669) (Table S1). There were small to moderate differences in sociodemographic variables between those who were in our analysis sample and those who were not, providing some evidence for selective attrition bias.

**Table S1**

*Descriptives of those who reported housing data and those who did not.*

|  | Women with < 50% missing questionnaire items for poor housing quality at enrolment (n = 9669) | Women with > 50% missing questionnaire items for poor housing quality at enrolment (n = 4702) | Comparison test statistic and p-value |
| --- | --- | --- | --- |
| Ethnicity | | | |
| White | 97.41% | 95.05% | χ^2^ = 93.42,  *p* < .001 |
| Non-White | 2.59% | 4.95% |  |
| n | 7648 | 2950 |  |
| Smoking at enrolment | | | |
| Never | 48.80% | 38.54% | χ^2^ = 333.32,  *p* < .001 |
| Until pregnancy | 29.05% | 26.98% |  |
| During pregnancy | 22.14% | 34.49% |  |
| n | 6908 | 2569 |  |
| Familial Depressive History | | | |
| Both parents | 1.86% | 1.42% | χ^2^ = 109.82,  *p <* .001 |
| One parent | 18.22% | 15.33% |  |
| None | 79.93% | 83.24% |  |
| n | 9669 | 4702 |  |
| Age at Poor Housing Quality | | | |
| Mean (SD) | 27.81 (4.90) | 26.34 (5.20) | t = 21.31, *p* < .001,  d = 0.42 |
| Min, Max | 15.00, 45.00 | 15.00, 45.00 |  |
| n | 8330 | 3931 |  |
| Housing Instability at Study Enrolment | | | |
| Mean (SD) | 1.71 (1.83) | 1.84 (1.94) | t = -5.13, *p <* .001,  d = -0.10 |
| Min, Max | 0.00, 28.00 | 0.00, 20.00 |  |
| n | 8141 | 3748 |  |
| Poor Neighbourhood Quality at Study Enrolment | | | |
| Mean (SD) | 4.93 (2.27) | 5.34 (2.43) | t = -13.08, *p* < .001,  d = -0.26 |
| Min, Max | 1.00, 13.00 | 1.00, 13.00 |  |
| n | 8022 | 3755 |  |
| SES Risk Factors | | | |
| Mean (SD) | 1.63 (0.87) | 1.86 (0.91) | t = -8.08, *p* < .001,  d = -0.26 |
| Min, Max | 0.60, 5.60 | 0.60, 5.20 |  |
| n | 5828 | 1120 |  |

- 1. **Poor Housing Quality**

Questionnaires contained items from validated and reliable indicators of housing quality ^3^ and from the UK ‘Decent Housing Standard’ ^4^. House size, facilities, decorations and feelings towards the home were reverse coded, to ensure higher scores indicated poorer housing quality. For example, higher scores of house size and facilities indicate smaller homes and fewer facilities.

House size was measured via the total number of rooms in the property. Facilities were measured by asking if the property had access to the following features or not, ‘Hot running water’, ‘Indoor WC’, ‘Bath’, ‘Shower’, ‘Garden or Yard’, ‘Balcony’, and ‘Double glazed windows’. Decorations measured whether the living room, bedrooms, kitchen, and other rooms had modifications made to them in the last year including new paint, wallpaper, carpet, or furniture. Temperature was measured via ‘During the coldest time of year, describe the temperature in your bedrooms and living room’ with responses including ‘Very cold’, ‘Cold’, ‘0/About right’, ‘Warm’, and ‘Very warm’. Temperature was coded as deviation from ‘0/About right’, with greater deviations contributing to poorer housing quality. Problems measured both the presence and severity of leaks, damp, condensation, and mould in the home with the following responses, ‘Very serious’, ‘Fairly serious’, ‘Not serious’, ‘No leak/damp/condensation’. Lastly, feelings towards the home were measured by asking ‘Taking everything into account, which of the following best describes your feelings about your home?’ with responses including ‘Very dissatisfied’, ‘Dissatisfied’, ‘Fairly satisfied’, and ‘Very satisfied’. These indicators include both person-centered and house-centered variables in housing. For example, person-centered variables include temperature and feelings towards the home, whereas house-centered variables include house size, facilities, and problems in the home.

**2 Results**

**Table S2**

*Estimated edge weights.*

|  | Problems | Facilities | Size | Decorations | Temperature | Feelings | Depressive symptoms | SES Risk Factors | Age |
| --- | --- | --- | --- | --- | --- | --- | --- | --- | --- |
| Problems | -- |  |  |  |  |  |  |  |  |
| Facilities | 0.086 | -- |  |  |  |  |  |  |  |
| Size | -0.053 | 0.157 | -- |  |  |  |  |  |  |
| Decorations | 0.107 | 0.091 | 0.111 | -- |  |  |  |  |  |
| Temperature | 0.035 | 0.021 | 0.030 | -0.027 | -- |  |  |  |  |
| Feelings | 0.196 | 0.132 | 0.166 | 0.005 | 0.040 | -- |  |  |  |
| Depressive symptoms | 0.064 | -0.014 | -0.024 | -0.022 | 0.017 | 0.115 | -- |  |  |
| SES Risk Factors | 0.021 | 0.080 | 0.151 | -0.029 | 0.050 | 0.128 | 0.205 | -- |  |
| Age | 0.018 | 0.054 | -0.300 | 0.161 | -0.058 | 0.017 | 0.014 | -0.152 | -- |

**Figure S1**


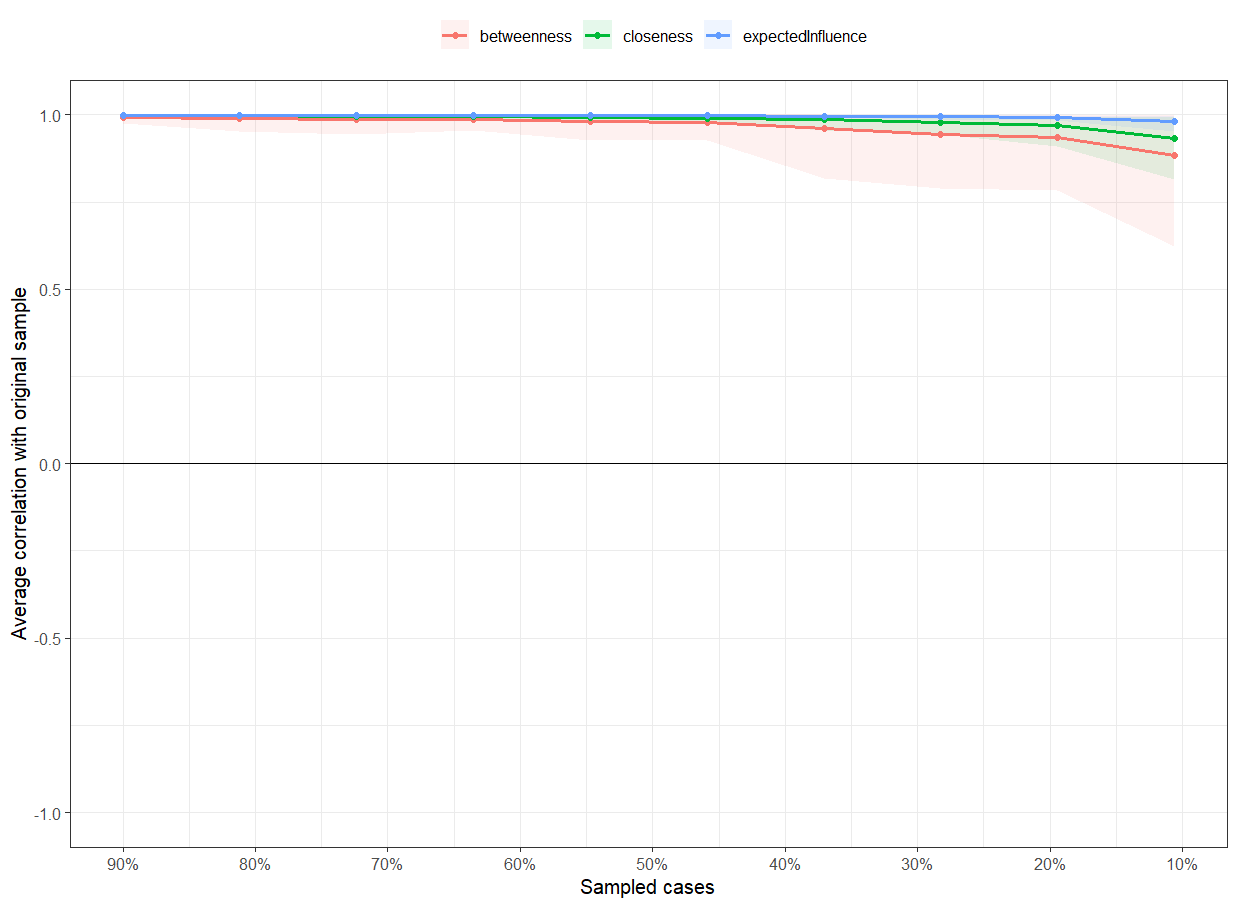


*Note.* This plot shows the centrality stability assessed through case-drop bootstrapping (1,000 resamples) down to 10% of the original sample. The lines represent the correlations of centrality scores (e.g., betweenness, closeness, and expected influence) with the original sample and 95% confidence intervals are represented by the shaded areas.

**Figure S2**

*
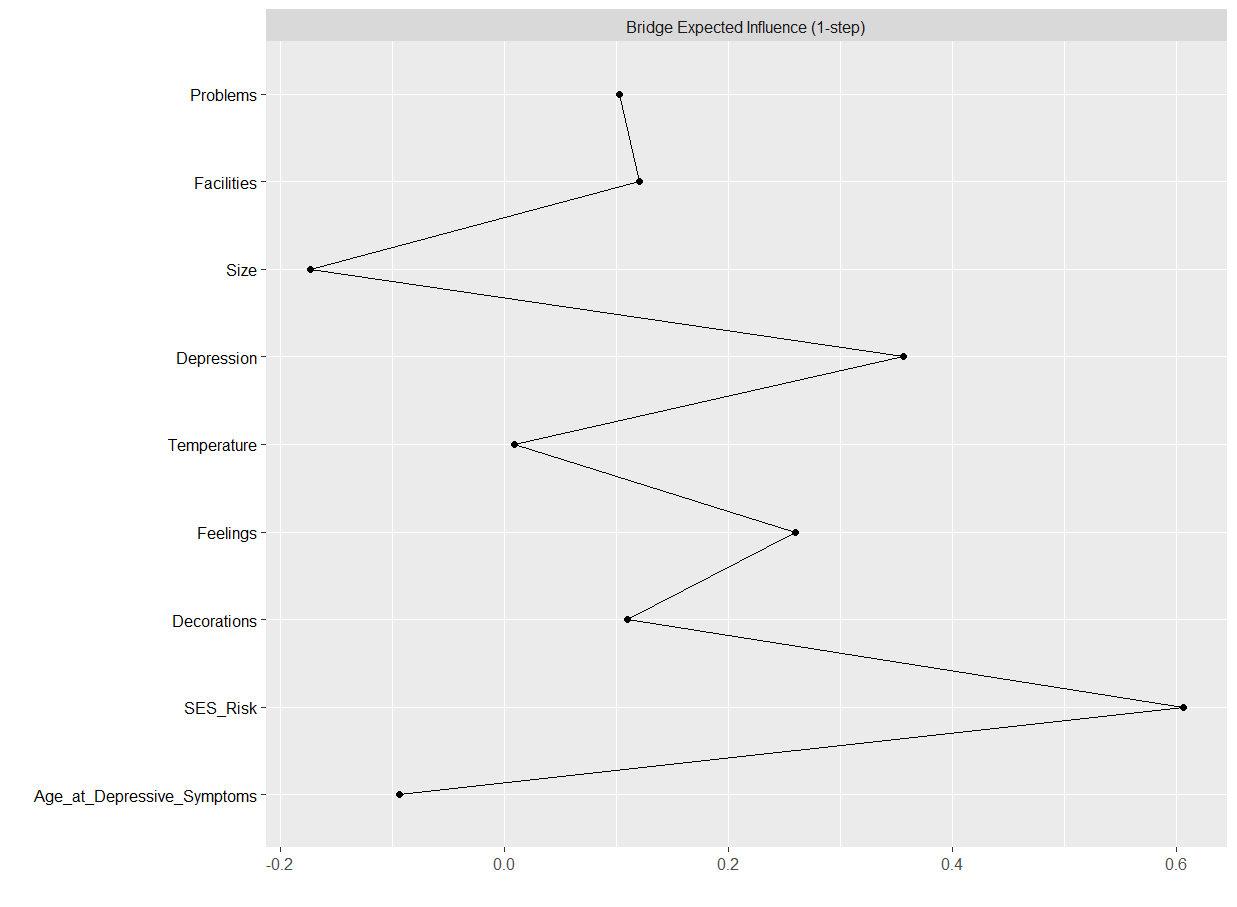
*

Bridge Expected Influence

*Note*. Bridge Expected Influence centrality measure for each poor housing quality indicator and depression.

**Table S3**

*Centrality values*

|  | Betweenness | Closeness | Expected Influence |
| --- | --- | --- | --- |
| Problems | 1.00 | 0.071 | 0.103 |
| Facilities | 0.00 | 0.065 | 0.121 |
| Size | 4.00 | 0.090 | -0.173 |
| Decorations | 6.00 | 0.071 | 0.110 |
| Temperature | 0.00 | 0.035 | 0.009 |
| Feelings | 3.00 | 0.081 | 0.260 |
| Depressive symptoms | 0.00 | 0.062 | 0.356 |
| SES Risk Factors | 5.00 | 0.087 | 0.606 |
| Age | 0.00 | 0.050 | -0.093 |

*Note.* Betweenness, Closeness, and Expected Influence centrality values (1-step) for each poor housing quality indicator and depression.

References

1. Sanders, F. *et al.* Home and Epigenome: DNA Methylation as a Link Between Poor Housing Quality and Depressive Symptoms. (2024).

2. Buuren, S. van & Groothuis-Oudshoorn, K. mice: Multivariate Imputation by Chained Equations in R. *J. Stat. Softw.* **45**, 1–67 (2011).

3. Evans, G. W., Wells, N. M., Chan, H. Y. & Saltzman, H. Housing quality and mental health. *J. Consult. Clin. Psychol.* **68**, 526–530 (2000).

4. Department for Levelling Up, Housing and Communities. A decent home: definition and guidance. *GOV.UK* https://www.gov.uk/government/publications/a-decent-home-definition-and-guidance (2006).
